# Accuracy and longitudinal reproducibility of quantitative femorotibial cartilage measures derived from automated U-Net-based segmentation of two different MRI contrasts: data from the osteoarthritis initiative healthy reference cohort

**DOI:** 10.1007/s10334-020-00889-7

**Published:** 2020-10-06

**Authors:** Wolfgang Wirth, Felix Eckstein, Jana Kemnitz, Christian Frederik Baumgartner, Ender Konukoglu, David Fuerst, Akshay Sanjay Chaudhari

**Affiliations:** 1 Department of Imaging and Functional Musculoskeletal Research, Institute of Anatomy and Cell Biology, Paracelsus Medical University Salzburg and Nuremberg, Strubergasse 21, 5020 Salzburg, Austria; 2 Ludwig Boltzmann Institute for Arthritis and Rehabilitation, Paracelsus Medical University, Salzburg, Austria; 3 Chondrometrics GmbH, Ainring, Germany; 4 ETH, Zurich, Switzerland; 5 Department of Radiology, Stanford University, Stanford, CA USA

**Keywords:** Cartilage, Automated segmentation, Knee osteoarthritis, Magnetic resonance imaging, Convolutional neural network

## Abstract

**Objective:**

To evaluate the agreement, accuracy, and longitudinal reproducibility of quantitative cartilage morphometry from 2D U-Net-based automated segmentations for 3T coronal fast low angle shot (corFLASH) and sagittal double echo at steady-state (sagDESS) MRI.

**Methods:**

2D U-Nets were trained using manual, quality-controlled femorotibial cartilage segmentations available for 92 Osteoarthritis Initiative healthy reference cohort participants from both corFLASH and sagDESS (*n* = 50/21/21 training/validation/test-set). Cartilage morphometry was computed from automated and manual segmentations for knees from the test-set. Agreement and accuracy were evaluated from baseline visits (dice similarity coefficient: DSC, correlation analysis, systematic offset). The longitudinal reproducibility was assessed from year-1 and -2 follow-up visits (root-mean-squared coefficient of variation, RMSCV%).

**Results:**

Automated segmentations showed high agreement (DSC 0.89–0.92) and high correlations (*r* ≥ 0.92) with manual ground truth for both corFLASH and sagDESS and only small systematic offsets (≤ 10.1%). The automated measurements showed a similar test–retest reproducibility over 1 year (RMSCV% 1.0–4.5%) as manual measurements (RMSCV% 0.5–2.5%).

**Discussion:**

The 2D U-Net-based automated segmentation method yielded high agreement compared with manual segmentation and also demonstrated high accuracy and longitudinal test–retest reproducibility for morphometric analysis of articular cartilage derived from it, using both corFLASH and sagDESS.

## Introduction

Osteoarthritis (OA) is a highly prevalent, chronic disease that affects more than 300 million people world-wide [1, 2]. OA patients experience pain and functional limitations, and the knee is by far the most commonly affected joint [2]. Amongst other structural pathologies of this whole-joint-disease, articular cartilage loss is a hallmark of knee OA. While radiography was previously used to assess the structural progression of OA, quantitative measurement of articular cartilage based on serial magnetic resonance images (MRI) is now the method of choice and provides the high test–retest precision and sensitivity to longitudinal change required for clinical trials [3–5]. The use of quantitative MRI also has revolutionized the conduct of clinical trials on structure or disease modifying OA drugs (S/DMOADs) [5–8], by having recently been scaled up from exploratory to secondary or even primary endpoints for submission for potential regulatory approval [6, 9].

Several groups have proposed semi- or fully automated approaches for reducing the time required for the segmentation of articular cartilage from MRI, including model-, atlas-, graph-, voxel classification-, or active-contour-based methods [10–12]. More recently, convolutional neural networks (CNNs), primarily based on the U-Net architecture [13], have been employed for automated cartilage segmentations and have demonstrated a good segmentation agreement between automated vs. ground-truth approaches [14–23]. Yet, only few of these CNN-based studies examined the accuracy of quantitative cartilage measures (e.g. thickness, volume, and surface area) derived from CNN-based segmentations [14, 16, 23]. Particularly, none of these reported the longitudinal stability or test–retest precision of quantitative cartilage measures derived from CNN-based cartilage segmentation, which is an important prerequisite before a segmentation methodology can be applied to data from a clinical trial, or compared the segmentation and analysis performance between different MRI sequences typically used in osteoarthritis studies [24].

The objective of the current study was, therefore, to evaluate the segmentation agreement as well as the accuracy and longitudinal test–retest reproducibility of quantitative cartilage measures obtained from a 2D U-Net-based methodology for automated femorotibial cartilage segmentation using two different MRI sequences for the same subject. To that end, we used data from the publicly accessible Osteoarthritis Initiative (OAI) cohort, specifically the subcohort of reference knees that were free of symptoms, signs and risk factors of knee OA, and for which cartilage thickness values (and their stability over time) have been reported previously [25–27]. Specifically, this work encompasses:

- Evaluating the agreement between automated and quality-controlled, manual segmentation of articular cartilage as “ground truth”.
- Testing the accuracy (correlations and systematic offsets) of quantitative cartilage morphometry measures (thickness, volume, surface areas) derived from automated segmentations compared to manual segmentation.
- Analysis of the longitudinal test–retest reproducibility of quantitative cartilage measures derived from automated vs. manual segmentation over a 1-year period (using year-1 and -2 follow-up data).
- Comparison of the agreement, accuracy, and longitudinal test–retest reproducibility of the automated segmentation (and quantitative cartilage measures derived therefrom) between two different MRI sequences with different contrasts and orientations.

## Materials and methods

### Participants and MR imaging

This study used data from the OAI (clinicaltrials.gov: NCT00080171) [28]. The OAI was approved by the Committee on Human Research, the Institutional Review Board for the University of California, San Francisco (UCSF). All OAI participants provided written informed consent, and this study was carried out in accordance with the OAI data user agreement. The OAI enrolled participants aged 45–79 years with established knee OA (progression cohort, *n* = 1390), with risk of developing OA (incidence cohort, *n* = 3284), and participants without signs, symptoms, or risk factors for developing OA (reference cohort, *n* = 122, based on the initial clinical site readings). Demographic, clinical and radiographic data, as well as MRIs were collected by four clinical sites at the baseline visit and each of the annual follow-up visits (https://data-archive.nimh.nih.gov/oai/). MRIs were acquired by the OAI using 3T Magnetom Trio scanners (Siemens Medical Solutions, Erlangen, Germany) and quadrature transmit/receive knee coils (USA Instruments, Aurora, OH) [28, 29]. The OAI imaging protocol included coronal fast low angle shot (FLASH) acquisitions with water excitation (in-plane resolution 0.3125 × 0.3125 mm, slice thickness 1.5 mm, flip angle 12°, echo time 7.6 ms, repetition time 20 ms) of the right knees, and sagittal double echo steady state (DESS) with water excitation of both knees (in-plane resolution 0.37 × 0.46 mm, interpolated to 0.37 × 0.37 mm, slice thickness 0.7 mm, flip angle 25°, echo time 4.7 ms, repetition time 16.3 ms) [29].

The current study included all 92 participants from the OAI reference cohort that were confirmed to be free from radiographic signs of OA in both of their knees during post hoc central readings by experienced readers [28], and that had at least the year-1 follow-up MRI available.

### Manual segmentation

Manual segmentations of the weight-bearing part of the femorotibial cartilages were available from previous projects for the right knees of the 92 OAI reference cohort participants [25–27]. Segmentations of baseline and year-1 follow-up MRIs from coronal FLASH (corFLASH) MRI were performed for all 92 right knees after the year-1 follow-up data from the OAI became available [25] and were later repeated together with year-2 and -4 follow-up MRIs for 81 of the 92 knees that also had year-4 follow-up MRIs available [26]. Segmentations of baseline, year-1, -2, and -4 follow-up sagittal DESS (sagDESS) MRIs were performed for the same 92 knees, and year-2/year-4 follow-up MRIs were available for 88/82 of the knees, respectively [26, 27].

**Fig. 1:**
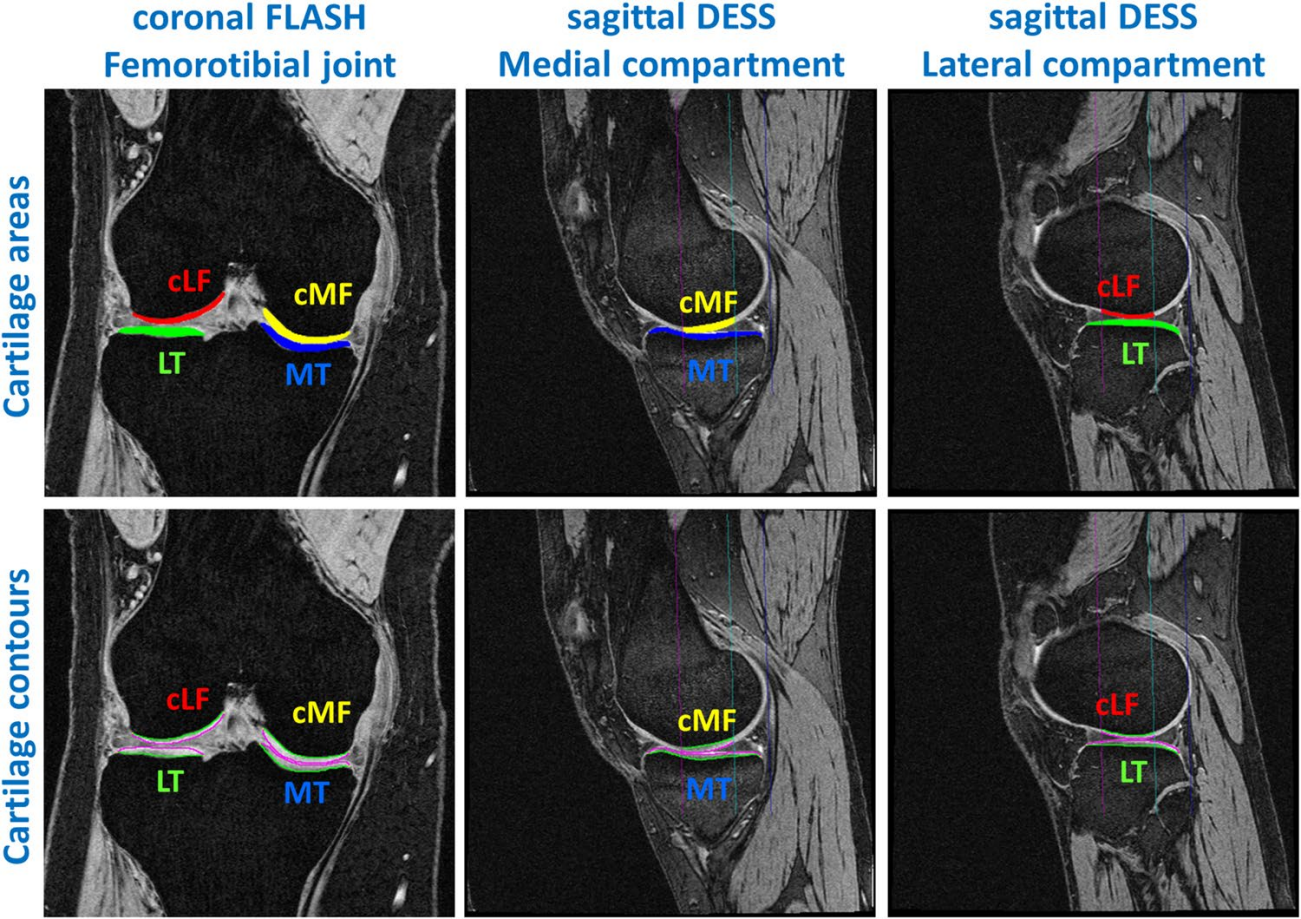
Manual segmentation of the femorotibial cartilages [*MT/LT* medial/lateral tibia, *cMF/cLF* central (weight-bearing) part of the medial and lateral femoral condyles] from coronal FLASH and sagittal DESS MRI. The figure shows the cartilage areas (top row) and the cartilage contours (bottom; green: total area of subchondral bone; magenta: cartilage surface area). The sagittal MRIs also show the 60% femoral region of interest (magenta line: anterior margin; blue line: posterior end of the condyles; turquoise line: 60% margin)

Segmentation comprised the entire medial and lateral tibia (MT/LT), and the central (weight-bearing) part of the medial and lateral femoral condyles (cMF/cLF), defined as 60% of the distance between the inter-condylar notch and the posterior end of the condyles (Fig. 1) [30, 31]. This 60% femoral region of interest (ROI) was necessary to avoid the inclusion of posterior parts of the cartilages in the segmentation, which are affected from partial volume effects in coronal MRIs and display a lesser amount of longitudinal change than the weight-bearing part in knee OA [30]. Manual segmentation was performed by a team of experienced readers using custom software (Chondrometrics GmbH, Ainring, Germany) by tracing the subchondral bone (tAB) and articular cartilage surface area (AC) of all four femorotibial cartilages (Fig. 1) [32]. All visits of each knee were segmented by the same reader, using one of the visits as a reference, but with blinding to the image dates, visit identifiers, and acquisition order. All manual segmentations were quality-controlled by an expert reader.

### Automated, U-Net-based segmentation

**Fig. 2:**
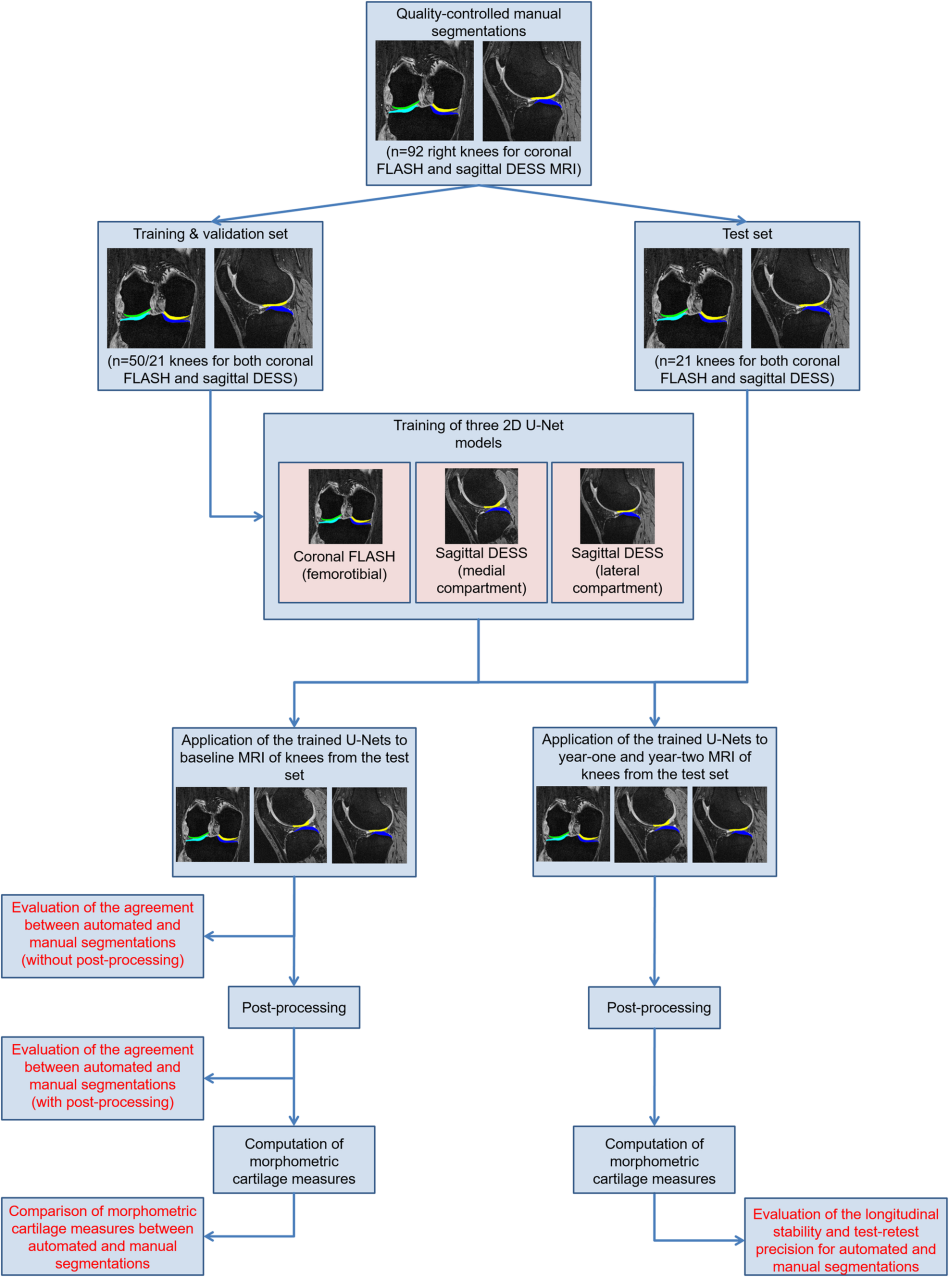
Overview over the workflow and analysis steps used for the current study

The 92 OAI reference cohort participants were divided into a training (*n* = 50), validation (*n* = 21) and test set (*n* = 21, Fig. 2). The division was controlled to ensure a similar distribution of sex and body height between the sets. Participants for which no manual year-2 segmentations from corFLASH MRI were available, were only considered for inclusion into the training and validation set, to ensure that manual segmentations from year-1 and -2 follow-up MRIs were available for all participants from the test set to evaluate the longitudinal test–retest reproducibility (see below).

The automated segmentation method was based on the 2D encoder-decoder U-Net architecture proposed by Ronneberger et al. [13] with the number of feature maps in the transpose convolutions of the up-sampling path set to the number of feature classes [33]. This implementation of the 2D U-Net architecture has been previously applied to the segmentation of MRIs of cardiac tissue [33] and thigh muscle cross-sectional areas [34]. In the current study, the U-Net was trained using a weighted cross-entropy loss function (background weight 1/[1 + 2 × number of feature classes]; foreground weight 2/[1 + 2 × number of feature classes]) that was minimized using the adaptive moment estimation (ADAM) optimizer (initial learning rate 0.01, decay rate 0.1, beta1 = 0.9, beta2 = 0.999) [35]. All network weights were randomly initialized using the tensorflow variance scaling initializer. The software was implemented in Python (Python Software Foundation, DE, USA) using the Tensorflow framework (Google LLC, CA, USA).

The training was performed on the training set using full-resolution (512 × 512 pixel for corFLASH, 384× 384 pixel for sagDESS), full-sized MRI slices on a NVIDIA RTX 2080TI GPU. The signal intensity was normalized in each slice by subtracting the mean intensity, and dividing by the standard deviation of the signal intensity. Bright voxels in the image corners (15 × 15 pixels) were set to zero intensity to avoid a negative impact of these imaging artefacts on the signal intensity normalization.

For corFLASH MRI, one network was trained comprising all four femorotibial cartilage plates. For sagDESS MRI, two networks were trained in parallel, one for the medial femorotibial compartment (MFTC) and one for the lateral femorotibial compartment (LFTC). Each cartilage plate was treated as an individual feature class, with the training including only the segmented slices. The three network weights (corFLASH, sagDESS MFTC, sagDESS LFTC) that achieved the best segmentation agreement with the validation set during 50 epochs were eventually applied for automatic segmentation of the test set (Fig. 2). The automated segmentations were not quality-controlled and not manually corrected.

### Automated post-processing

Because the predictions made by the U-Net may extend into anatomically implausible locations and because the automated segmentations required adaption before the computation of morphometric cartilage measures, the following, automated rule-based post-processing steps were implemented as a non-interactive command line program using C++:

- Filling of small gaps by detecting enclosed, unsegmented areas
- Removal of segmentations in slices not connected to the segmentation in the same or other slices (i.e., segmentations at implausible locations)
- Removal of spikes (smoothing)
- Removal of femoral cartilage segmentations outside the (60%) femoral ROI
- Separation of segmentations into the subchondral bone area (tAB), articular cartilage surface area (AC), and inner cartilage (IC), which was required for the computation of morphological parameters (see below).

The separation of segmentations into tAB, AC, and IC was performed for each structure and each slice separately by identifying the two points with the greatest distance from each other and by subsequently assigning the border pixel of the structures’ segmentation to the tAB and AC. When segmentation of the cMF and cLF bordered the femoral ROI (sagDESS only), the intersection between the segmentation and the femoral ROI was used instead. Non-border voxels were assigned to IC.

### Statistical analysis

The agreement between automated and quality-controlled manual segmentations was evaluated using the 3D Dice similarity coefficient (DSC), the 3D volume overlap error (VOE), the 3D Hausdorff distance (HD), and the 3D average symmetric surface distance (ASSD) for the knees from the test set, before and after applying the post-processing steps.

To evaluate the repeatability of the training process, training was repeated from scratch using the same sets with a different random initialization of network weights (“repeated run”). To evaluate, whether the agreement between automated vs. manual segmentations is dependent on the assignment of knees to each of the sets, training was repeated using the knees from the validation and test set, together with 8 knees from the original training set as training set, and by assigning the remaining 42 knees from the training set into validation and test sets (each *n* = 21, “reversed run”). For both these runs, the agreement of automated vs. manual segmentations was again evaluated using the DSC, VOE, HD, and ASSD, after automatically segmenting the cartilages from the respective test set (with and without post-processing).

Cartilage thickness, cartilage volume, and the total area of subchondral bone (tAB) were calculated from both manual and post-processed automated segmentations of the knees in the test set using custom software (Chondrometrics GmbH, Ainring, Germany). Measures for the medial and lateral femorotibial compartment (MFTC/LFTC) were calculated as sums of MT + cMF and LT + cLF, respectively. The accuracy of baseline cartilage measures computed from the automated segmentations vs. measures computed from manual segmentations was evaluated by examining the Pearson correlation. In addition, paired *t* tests were used to assess differences between cartilage measures computed from automated vs manual segmentations. Bland and Altman plots were used to evaluate potential systematic offsets between both segmentation methods, and between corFLASH and sagDESS. Furthermore, Pearson correlation analyses were conducted to study the association of cartilage thickness differences between automated and manual segmentations vs. DSC, VOE, HD, and ASSD values.

Cartilage thickness has been previously observed to remain stable over periods of 1 year and longer in knees from the OAI reference cohort [25, 26]. Consequently, the year-1 and -2 follow-up visits were used to assess the longitudinal test–retest reproducibility of the automated cartilage analysis over such an observation period typical of interventional clinical trials. The longitudinal stability was assessed using a paired *t* test and the test–retest reproducibility using the root-mean-square standard deviation (RMSSD) and coefficient of variation (RMSCV%) of repeated measurements. To quantitatively evaluate whether the trained CNNs were overfitting to the data used during the training (training and validation sets), the longitudinal test–retest reproducibility was additionally computed for the knees from the validation and training set that had year-1 and -2 follow-up visits available (*n* = 19/41 pairs). The standard error of the measurement (SEM) and the smallest detectable change (SDC) threshold were calculated from year-1 and -2 data for the knees in the test set as described previously [36].

Demographic variables were compared between groups using unpaired *t* tests. The significance level for all statistical testing was set to *α* = 0.05. Descriptive statistics and *t* tests were computed using Excel 2010 (Microsoft Corporation, WA, USA).

## Results

The 55 female and 37 male OAI reference cohort participants were on average 54.7 ± 7.5 years old, had a BMI of 24.4 ± 3.1 kg/m^2^ and a body height of 1.68 ± 0.09 m (Table 1). These demographic data did not differ statistically significantly between training, validation, and test set (*p* ≥ 0.15).

**Table 1:** Demographic data

	Training set (*n* = 50)		Validation set (*n* = 21)		Test set (*n* = 21)	
	*N*/Mean	%/SD	*N*/Mean	%/SD	*N*/Mean	%/SD
Sex						
Women	29	58	13	62	13	62
Men	21	42	8	38	8	38
Age (years)	54.1	7.3	53.9	8.0	56.9	7.5
BMI (kg/m^2^)	24.1	3.0	24.5	3.2	25.1	3.3
Height (m)	1.68	0.09	1.67	0.09	1.67	0.09

*SD* standard deviation

During the training of the networks, the best segmentation agreement with data from the validation set was achieved for corFLASH/sagDESS LFTC/sagDESS MFTC after 14/33/34 epochs (99/167/159 min of training), and these U-Net weights were subsequently chosen for the automated segmentations on the hold-out test set.

### Agreement of the automated U-Net segmentation with manual segmentation

**Fig. 3:**
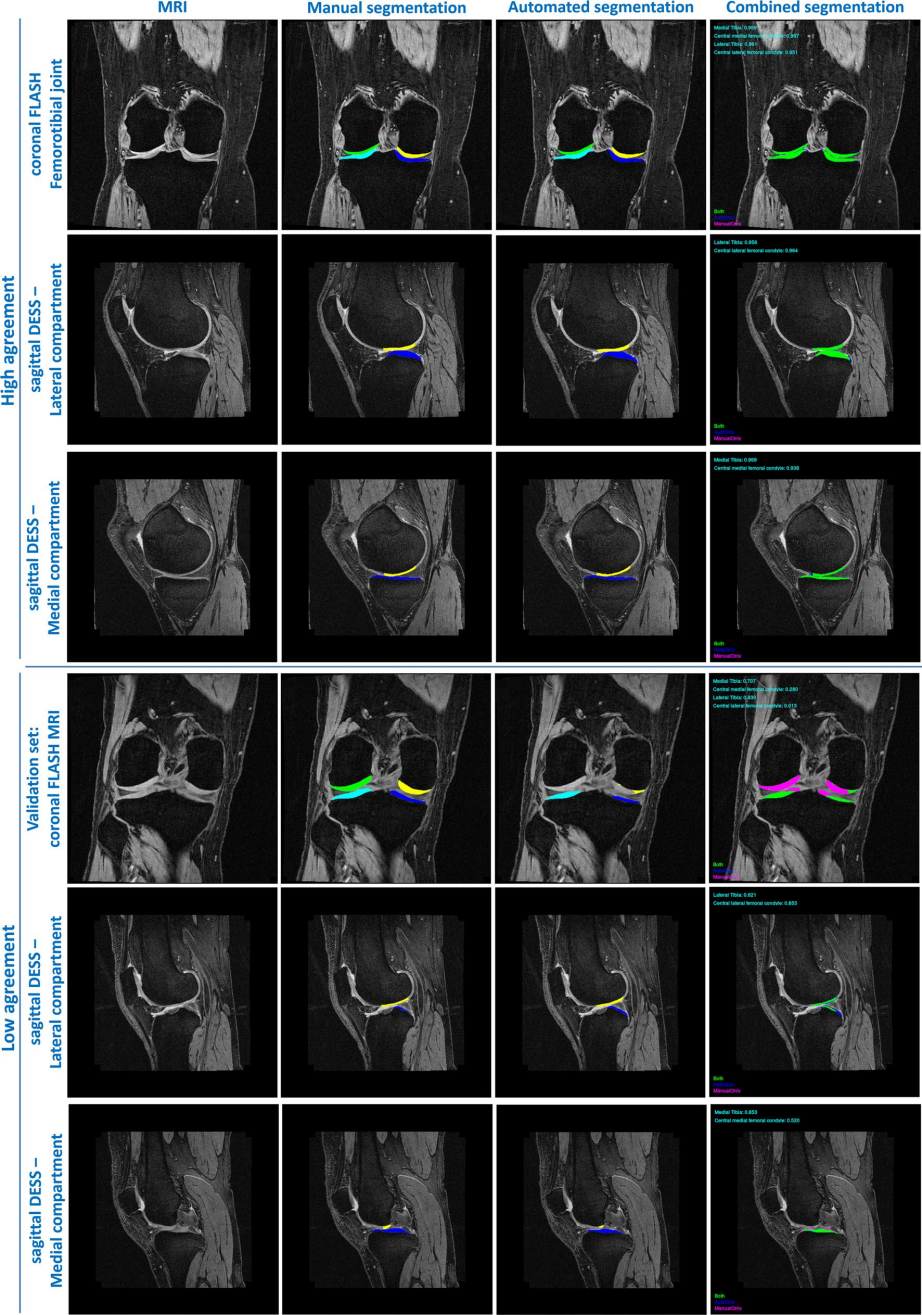
Examples of manual and U-Net-based automated segmentations from coronal FLASH and sagittal DESS illustrating the range of agreement observed in this study. Rows 1–3: Examples with high agreement, rows 4–6: examples with low agreement or segmentation errors taken from the training and validation set. Cartilage plates are shown in blue (medial tibia), yellow (central medial femur), turquoise (lateral tibia), and green (central lateral femur) in the two middle columns. The right column shows pixel contained in both manual and automated segmentations in green, pixel only contained in manual segmentations in purple, and pixel only contained in automated segmentations in blue

A high agreement was observed between automated and manual cartilage segmentations for both corFLASH and sagDESS MRI already before the post-processing (Table 2). The DSC ranged from 0.88 ± 0.03 to 0.92 ± 0.02, the VOE from 14.9 ± 3.3 to 21.9 ± 4.8%, the HD from 2.8 ± 1.1 to 8.3 ± 13.3 mm, and the ASSD from 0.13 ± 0.03 to 0.28 ± 0.13 mm (Table 2). Post-processing only had a small effect on the DSC (range 0.89 ± 0.03–0.92 ± 0.02) and the VOE (range 14.9 ± 3.3–20.1 ± 4.4%), but notably reduced the HD (range 2.1 ± 0.6–3.2 ± 0.9 mm) and the ASSD (range 0.13 ± 0.03–0.17 ± 0.06 mm, Table 2, Fig. 3).

**Table 2:** Agreement between manual and U-Net-based automated segmentations determined from *n* = 21 knees in the test set in the primary run (top) and the repeated run (bottom)

	DSC				VOE (%)				HD (mm)				ASSD (mm)			
	corFLASH		sagDESS		corFLASH		sagDESS		corFLASH		sagDESS		corFLASH		sagDESS	
	Mean	SD	Mean	SD	Mean	SD	Mean	SD	Mean	SD	Mean	SD	Mean	SD	Mean	SD
*Main run: before post-processing*																
MT	0.92	0.02	0.91	0.02	15.5	3.0	17.1	2.7	8.27	13.32	2.79	1.08	0.15	0.04	0.13	0.03
cMF	0.88	0.03	0.89	0.03	21.9	4.8	20.0	4.3	5.72	11.02	6.14	11.24	0.28	0.13	0.18	0.07
LT	0.92	0.02	0.92	0.02	14.9	3.3	15.4	2.9	8.06	8.80	5.31	8.46	0.17	0.05	0.17	0.04
cLF	0.88	0.02	0.90	0.02	20.8	3.8	17.8	2.8	3.66	2.33	3.86	5.54	0.26	0.09	0.14	0.03
*Main run: after post-processing*																
MT	0.92	0.02	0.91	0.02	15.5	3.0	17.0	2.7	2.28	0.67	2.39	0.47	0.14	0.03	0.13	0.03
cMF	0.91	0.03	0.89	0.03	16.4	4.6	20.1	4.4	2.60	1.21	3.36	0.72	0.13	0.08	0.17	0.06
LT	0.92	0.02	0.92	0.02	14.9	3.3	15.4	2.9	3.00	1.09	3.21	0.92	0.16	0.04	0.17	0.04
cLF	0.91	0.02	0.90	0.02	15.8	4.0	17.9	2.8	2.08	0.56	2.65	0.55	0.13	0.06	0.14	0.03
*Repeated run: before post-processing*																
MT	0.92	0.02	0.91	0.02	15.3	2.7	16.4	2.8	6.38	8.39	2.52	0.78	0.14	0.03	0.13	0.03
cMF	0.84	0.03	0.89	0.03	26.8	5.2	20.0	4.5	8.70	10.12	8.21	11.86	0.44	0.21	0.18	0.09
LT	0.92	0.02	0.92	0.02	14.8	3.4	15.0	2.8	5.64	7.24	3.00	1.00	0.15	0.04	0.16	0.03
cLF	0.88	0.03	0.91	0.02	21.2	4.7	17.0	2.6	5.63	8.64	7.15	12.77	0.25	0.12	0.14	0.05
*Repeated run: after post-processing*																
MT	0.92	0.02	0.91	0.02	15.2	2.7	16.4	2.8	2.21	0.68	2.30	0.62	0.14	0.03	0.13	0.03
cMF	0.91	0.02	0.89	0.03	17.0	3.9	20.2	4.6	2.50	1.00	3.40	0.75	0.14	0.06	0.18	0.07
LT	0.92	0.02	0.92	0.02	14.7	3.4	15.0	2.8	3.02	0.93	3.00	1.00	0.15	0.04	0.17	0.03
cLF	0.90	0.03	0.91	0.02	17.5	4.8	17.1	2.5	2.18	0.48	2.42	0.49	0.16	0.08	0.13	0.02

Agreement was assessed from coronal FLASH (corFLASH) and sagittal DESS (sagDESS) MRI acquired at the OAI baseline visit

*DSC* dice similarity coefficient, *VOE* volume overlap error, *HD* Hausdorff distance, *ASSD* average symmetric surface distance, *MT/LT* medial/lateral tibia, *cMF/cLF* central medial/lateral femoral condyle

The agreement between automated vs. manual segmentation obtained in both the repeated run (Table 2) and the reversed run (data now shown) was largely consistent with the results from the main run: A somewhat lower agreement was observed for the cMF in the repeated run for corFLASH MRI (Table 2). A similar observation was made for the cMF (DSC 0.86 ± 0.04, VOE 23.9 ± 5.6%, HD 16.5 ± 16.3 mm, ASSD 0.37 ± 0.20 mm) and cLF (DSC 0.84 ± 0.05, VOE 26.8 ± 7.0%, HD 4.1 ± 13 mm, ASSD 0.40 ± 0.19 mm) with corFLASH MRI in the reversed run (data not shown). These differences were only evident prior to the post-processing.

### Accuracy of cartilage morphometry using automated, U-Net vs. manual segmentation

**Fig. 4:**
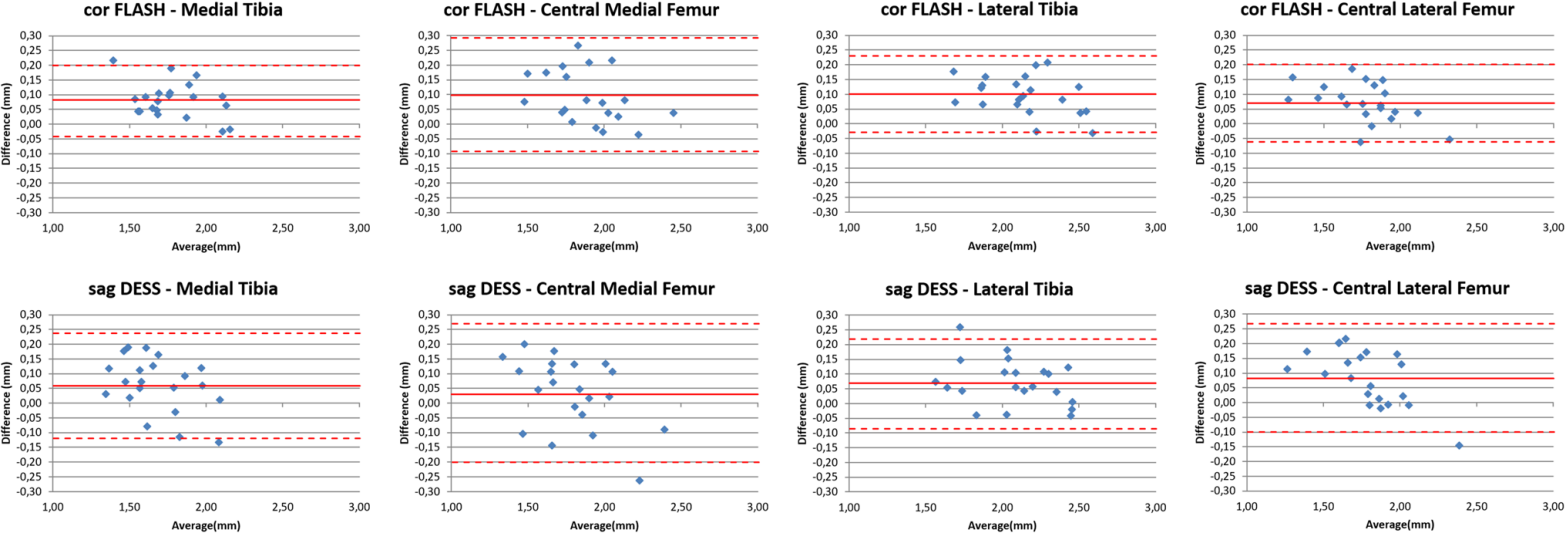
Bland and Altman plots relating the cartilage thickness difference between U-Net-based automated vs. manual segmentations to the cartilage thickness averaged over these two segmentation methods for coronal FLASH MRI (corFLASH, top row) and sagittal DESS MRI (sagDESS, bottom row). The mean difference (continuous line) and the 95% limits of agreement (dotted lines) are shown in red for each of the four femorotibial cartilages

**Fig. 5:**
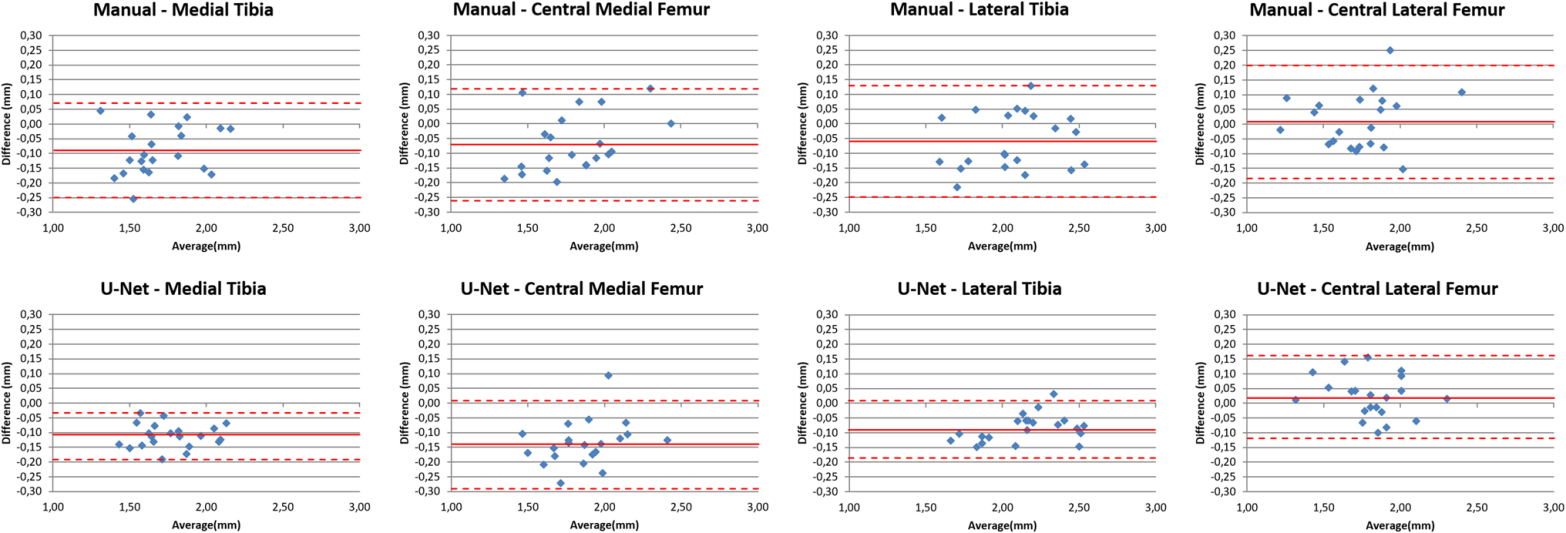
Bland and Altman plots relating the cartilage thickness difference between coronal FLASH vs sagittal DESS MRI to the cartilage thickness averaged over these two imaging protocols for both manual (top row) and U-Net-based automated segmentations (bottom row). The mean difference (continuous line) and the 95% limits of agreement (dotted lines) are shown in red for each of the four femorotibial cartilages

All morphometric cartilage measures computed from the automated segmentations of the baseline MRIs in the test set displayed high correlations with those obtained from manual segmentations (range *r* = 0.92–0.99, Table 3). Cartilage thickness from the automated segmentation had a slight, but consistent overestimation when compared to the measures derived from manual segmentation for both corFLASH and sagDESS (range 1.9–5.5%, Table 3). This difference was statistically significant in all cartilage plates, except for the cMF with sagDESS (Table 3). Bland and Altman plots comparing cartilage thickness measures computed from automated vs. manual segmentations are shown in Fig. 4, Bland and Altman plots comparing cartilage thickness between corFLASH and sagDESS MRI in Fig. 5. In brief, cartilage thickness computations were highly consistent between corFLASH and sagDESS using both methodological approaches. The mean difference tended to be closer to zero for the manual than for the automated segmentations, whereas the limits of agreement tended to be narrower for the automated than the manual segmentations.

**Table 3:** Comparison of quantitative cartilage measures between manual and U-Net-based automated segmentations determined from *n* = 21 knees from the test set

		Manual		U-Net		Manual vs. U-Net		
		Mean	SD	Mean	SD	Diff (%)	*P*	*r*
*Cartilage thickness* (*mm*)								
MFTC	corFLASH	3.6	0.4	3.8	0.4	5.1	< 0.001	0.96
	sagDESS	3.4	0.5	3.5	0.4	2.8	0.019	0.95
MT	corFLASH	1.7	0.2	1.8	0.2	4.7	< 0.001	0.97
	sagDESS	1.6	0.2	1.7	0.2	3.7	0.006	0.93
cMF	corFLASH	1.8	0.3	1.9	0.2	5.5	< 0.001	0.93
	sagDESS	1.8	0.3	1.8	0.2	1.9	0.224	0.92
LFTC	corFLASH	3.8	0.5	4.0	0.5	4.4	< 0.001	0.98
	sagDESS	3.8	0.5	3.9	0.5	4.1	< 0.001	0.97
LT	corFLASH	2.1	0.3	2.2	0.3	4.6	< 0.001	0.97
	sagDESS	2.0	0.3	2.1	0.3	3.5	< 0.001	0.97
cLF	corFLASH	1.7	0.3	1.8	0.2	4.2	< 0.001	0.97
	sagDESS	1.7	0.3	1.8	0.2	4.8	0.001	0.96
*Cartilage volume* (*mm*^*3*^)								
MFTC	corFLASH	2947	829	3218	797	9.2	< 0.001	0.99
	sagDESS	2801	794	2912	737	4.0	0.005	0.98
MT	corFLASH	1945	570	2120	534	9.0	< 0.001	0.99
	sagDESS	1703	532	1848	496	8.5	< 0.001	0.98
cMF	corFLASH	1003	279	1099	277	9.6	< 0.001	0.96
	sagDESS	1098	285	1064	256	-3.1	0.154	0.93
LFTC	corFLASH	3123	859	3421	904	9.6	< 0.001	0.99
	sagDESS	3291	928	3433	872	4.3	< 0.001	0.99
LT	corFLASH	2083	589	2294	622	10.1	< 0.001	0.99
	sagDESS	2144	646	2295	618	7.0	< 0.001	0.99
cLF	corFLASH	1039	298	1128	302	8.5	< 0.001	0.98
	sagDESS	1147	311	1138	264	-0.8	0.623	0.97
*Total area of subchondral bone* (*cm*^*2*^)								
MFTC	corFLASH	16.1	2.8	16.3	2.8	1.5	0.040	0.98
	sagDESS	15.3	2.7	15.7	2.7	2.5	< 0.001	0.99
MT	corFLASH	11.0	1.9	11.2	1.8	1.7	0.027	0.98
	sagDESS	10.0	1.7	10.4	1.8	3.8	< 0.001	0.99
cMF	corFLASH	5.1	1.0	5.2	1.0	1.1	0.395	0.96
	sagDESS	5.3	1.0	5.3	1.0	0.0	0.999	0.95
LFTC	corFLASH	15.2	2.6	15.5	2.7	1.9	0.015	0.98
	sagDESS	15.6	2.8	15.8	2.7	1.6	0.024	0.99
LT	corFLASH	9.4	1.6	9.7	1.7	2.9	0.010	0.96
	sagDESS	9.9	1.7	10.1	1.7	2.2	0.018	0.98
cLF	corFLASH	5.8	1.1	5.8	1.1	0.1	0.904	0.98
	sagDESS	5.7	1.1	5.7	1.0	0.5	0.485	0.99

Quantitative measures were calculated from coronal FLASH (corFLASH) and sagittal DESS (sagDESS) MRI acquired at the OAI baseline visit

*MFTC/LFTC* medial/lateral femorotibial compartment, *MT/LT* medial/lateral tibia, *cMF/cLF* central medial/lateral femoral condyle, *P p* value from paired *t*-tests, *r* Pearson correlation coefficient

Cartilage volume also was statistically significantly greater when determined from automated vs. manual segmentation (range − 3.1–10.1%), except for the cMF and cLF in the sagDESS (Table 3). The total area of subchondral bone (tAB) was significantly greater when determined from automated vs. manual segmentation for the tibial cartilages (range 1.7–3.8%), whereas no significant differences were observed for the femoral condyles (range 0.0–1.1%, Table 3).

**Fig. 6:**
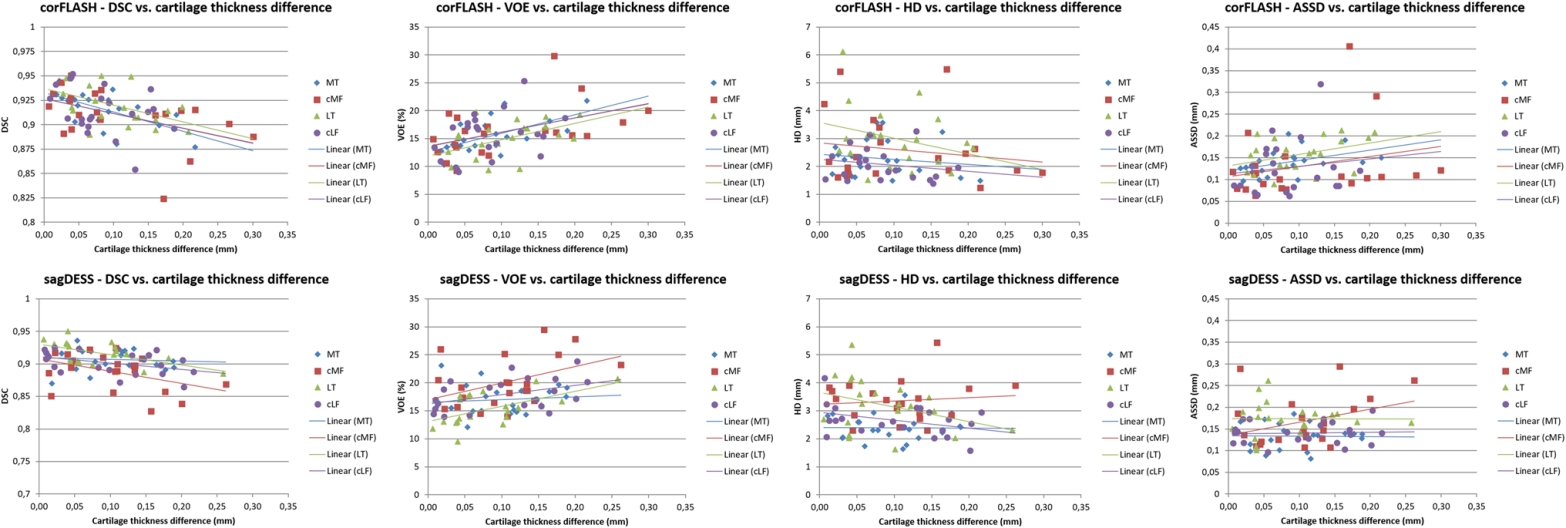
Scatter plots relating the agreement to the absolute difference in cartilage thickness between U-Net-based automated vs. manual segmentations for coronal FLASH MRI (corFLASH, top row) and sagittal DESS MRI. *sagDESS* bottom row, *DSC* dice similarity coefficient, *VOE* volume overlap error, *HD* Hausdorff distance, *ASSD* average symmetric surface distance, *MT/LT* medial/lateral tibia, *cMF/cLF* central medial/lateral femoral condyle

The DSC and VOE were significantly correlated with absolute cartilage thickness differences in the MT, cMF, and LT with corFLASH and for the LT with sagDESS (Table 4, Fig. 6). No statistically significant correlation was observed for the HD, but the ASSD was significantly correlated with thickness differences in the MT with corFLASH MRI (Table 4, Fig. 6).

**Table 4:** Correlation between absolute differences in cartilage thickness and measures of agreement between U-Net and manual segmentations

	DSC	VOE	HD	ASSD
*corFLASH*				
MT	− **0.61**	**0.61**	− 0.15	**0.44**
cMF	− **0.49**	**0.50**	− 0.17	0.26
LT	− **0.50**	**0.50**	− 0.29	0.38
cLF	− 0.32	0.32	− 0.19	0.14
*sagDESS*				
MT	− 0.09	0.10	− 0.02	− 0.01
cMF	− 0.42	0.43	0.10	0.33
LT	− **0.58**	**0.58**	− 0.35	0.00
cLF	− 0.42	0.42	− 0.38	0.05

Absolute cartilage thickness differences between U-Net-based, automated and manual segmentations calculated from coronal FLASH (corFLASH) and sagittal DESS (sagDESS) MRI acquired at the OAI baseline visit

*MT/LT* medial/lateral tibia, *cMF/cLF* central medial/lateral femoral condyle, *DSC* dice similarity coefficient, *HD* Hausdorff distance, *ASSD* average symmetric surface distance

Bold face indicates significant correlation coefficients (*p* < 0.05)

### Longitudinal test–retest reproducibility

The longitudinal change between year-1 and -2 follow-up observed in the 21 test set knees was between − 2.0 and 1.1% for automated and between − 0.9 and 1.6% for manual segmentations, with some of these changes reaching statistical significance, in particular with sagDESS MRI (Table 5).With corFLASH, the RMS SD for cartilage thickness ranged from 0.03 to 0.05 mm for manual, and from 0.02 to 0.06 mm for automated segmentations, with an RMS CV of 1.2–1.9% for manual and an RMS CV of 1.0–2.1% for automated segmentations (Table 5). With sagDESS, the RMS SD ranged from 0.03 to 0.05 mm for manual and automated segmentations, with an RMS CV of 1.2–2.0% for manual, and an RMS CV of 1.3–2.2% for automated segmentations (Table 5). Precision errors for cartilage volume and the total area of subchondral bone are also shown in Table 5.

**Table 5:** Longitudinal test–retest reproducibility for manual and U-Net-based segmentations determined from year-1 and -2 follow-up MRIs of *n* = 21 knees from the test set

	Manual					U-Net				
	MC ± SD	MC (%)	*P*	RMS SD	RMS CV%	MC ± SD	MC (%)	*P*	RMS SD	RMS CV%
*corFLASH*										
Cartilage thickness (mm)										
MFTC	0.00 ± 0.06	0.1	0.76	0.04	1.2	0.00 ± 0.05	0.0	0.93	0.04	1.0
MT	0.00 ± 0.04	0.0	0.96	0.02	1.5	0.01 ± 0.03	0.3	0.41	0.02	1.1
cMF	0.00 ± 0.04	0.2	0.67	0.03	1.5	0.00 ± 0.04	− 0.2	0.62	0.03	1.4
LFTC	0.01 ± 0.07	0.2	0.71	0.05	1.3	0.01 ± 0.09	0.2	0.64	0.06	1.6
LT	0.00 ± 0.04	− 0.2	0.72	0.03	1.3	0.01 ± 0.07	0.3	0.65	0.05	2.1
cLF	0.01 ± 0.04	0.5	0.38	0.03	1.9	0.00 ± 0.05	0.2	0.81	0.04	2.1
Cartilage volume (mm^3^)										
MFTC	2.6 ± 63.4	0.1	0.85	43.8	1.5	− 0.7 ± 79.3	0.0	0.97	54.7	1.7
MT	− 2.3 ± 51.6	− 0.1	0.84	35.6	1.9	5.5 ± 66.7	0.3	0.71	46.2	2.2
cMF	4.9 ± 30.2	0.5	0.47	21.1	2.2	− 6.3 ± 39.5	− 0.6	0.47	27.6	2.6
LFTC	− 9.2 ± 69.5	− 0.3	0.55	48.4	1.6	− 41.8 ± 108.1	− 1.2	0.09	80.2	2.4
LT	− 13.5 ± 58.9	− 0.6	0.31	41.8	2.0	− 33.1 ± 80.2	− 1.5	0.07	60.1	2.7
cLF	4.3 ± 30.4	0.4	0.52	21.2	2.1	− 8.6 ± 55.9	− 0.8	0.49	39.1	3.5
Total area of subchondral bone (cm^2^)										
MFTC	− 0.01 ± 0.12	− 0.1	0.66	0.08	0.5	− 0.03 ± 0.31	− 0.2	0.66	0.22	1.3
MT	− 0.03 ± 0.10	− 0.3	0.20	0.07	0.6	0.00 ± 0.23	0.0	1.00	0.16	1.4
cMF	0.02 ± 0.08	0.3	0.37	0.06	1.1	− 0.03 ± 0.17	− 0.6	0.43	0.12	2.4
LFTC	− 0.03 ± 0.12	− 0.2	0.33	0.09	0.6	− 0.17 ± 0.45	− 1.1	0.09	0.33	2.2
LT	− 0.02 ± 0.10	− 0.3	0.30	0.07	0.8	− 0.10 ± 0.38	− 1.0	0.25	0.27	2.8
cLF	0.00 ± 0.07	− 0.1	0.86	0.05	0.9	− 0.08 ± 0.24	− 1.3	0.16	0.17	3.0
*sagDESS*										
Cartilage thickness (mm)										
MFTC	0.03 ± 0.05	0.8	0.01	0.04	1.2	0.01 ± 0.07	0.3	0.47	0.05	1.4
MT	0.02 ± 0.03	1.1	0.02	0.03	1.5	0.02 ± 0.04	1.0	0.05	0.03	1.7
cMF	0.01 ± 0.04	0.6	0.22	0.03	1.7	− 0.01 ± 0.05	− 0.3	0.63	0.04	2.1
LFTC	0.01 ± 0.07	0.4	0.39	0.05	1.4	− 0.02 ± 0.07	− 0.4	0.33	0.05	1.3
LT	0.02 ± 0.06	0.8	0.24	0.04	2.0	− 0.01 ± 0.05	− 0.4	0.38	0.03	1.5
cLF	0.00 ± 0.04	− 0.1	0.90	0.03	1.6	− 0.01 ± 0.06	− 0.4	0.61	0.04	2.2
Cartilage volume (mm^3^)										
MFTC	23.9 ± 63.8	0.9	0.10	47.1	1.7	− 4.0 ± 79.5	− 0.1	0.82	54.9	1.9
MT	22.9 ± 44.5	1.3	0.03	34.7	2.0	3.3 ± 55.6	0.2	0.79	38.4	2.1
cMF	1.1 ± 34.3	0.1	0.89	23.7	2.2	− 7.3 ± 47.3	− 0.7	0.49	33.0	3.3
LFTC	34.3 ± 83.2	1.0	0.07	62.3	1.9	− 29.3 ± 123.5	− 0.9	0.29	87.7	2.7
LT	29.0 ± 70.6	1.4	0.07	52.9	2.5	− 5.4 ± 84.8	− 0.2	0.77	58.7	2.6
cLF	5.3 ± 28.8	0.5	0.41	20.2	1.7	− 23.9 ± 63.0	− 2.3	0.10	46.6	4.5
Total area of subchondral bone (cm^2^)										
MFTC	− 0.02 ± 0.24	− 0.1	0.77	0.16	1.1	− 0.13 ± 0.41	− 0.8	0.17	0.30	1.9
MT	0.04 ± 0.17	0.4	0.36	0.12	1.2	− 0.09 ± 0.30	− 0.8	0.21	0.22	2.1
cMF	− 0.05 ± 0.10	− 1.0	0.03	0.08	1.4	− 0.04 ± 0.27	− 0.8	0.48	0.19	3.6
LFTC	0.06 ± 0.17	0.4	0.10	0.13	0.8	− 0.12 ± 0.45	− 0.8	0.24	0.32	2.0
LT	0.05 ± 0.13	0.5	0.08	0.09	0.9	0.00 ± 0.27	0.0	0.96	0.19	1.9
cLF	0.02 ± 0.12	0.3	0.56	0.08	1.4	− 0.12 ± 0.25	− 2.1	0.04	0.19	3.4

Longitudinal stability and test–retest precision assessed from coronal FLASH (corFLASH) and sagittal DESS (sagDESS) MRI acquired at the OAI year-1 and -2 follow-up visits

*MC* mean change; *SD* standard deviation; *P p*-value from paired *t*-tests; *RMS SD* Root mean square standard deviation in mm (cartilage thickness), mm^3^ (cartilage volume), cm^2^ (total area of subchondral bone); *RMS CV* root mean square coefficient of variation (in %); *MFTC/LFTC* medial/lateral femorotibial compartment; *MT/LT* medial/lateral tibia; *cMF/cLF* central medial/lateral femoral condyle

Test–retest precision errors for evaluating the potential effect of network overfitting between year-1 and -2 follow-up MRIs were computed for 39 of the 41 knees from the training set, and for all 19 knees from the validation set that had manual year-1 and -2 MRI segmentations. In two of the knees from the training set, the computation of morphometric cartilage measures failed because of invalid segmentations that could not be corrected by the post-processing steps (Fig. 3). The test–retest precision errors observed in the training and validation sets were similar to those observed in knees from the test set, but tended to be greater for some of the parameters when computed from automated segmentations (data not shown), in particular for knees from the validation set with corFLASH. This can be attributed to three of the knees in the validation set, in which the automated segmentation from corFLASH differed notably between year-1 and -2 follow-up due to obvious segmentation errors (Fig. 3).

The standard error of the measurement (SEM) and the smallest detectable change (SDC) for cartilage thickness were comparable between automated (range SEM 0.04–0.13 mm; range SDC 0.11–0.36 mm) and manual (range SEM 0.05–0.10 mm; range SDC 0.13–0.29 mm) segmentations and between corFLASH (range SEM 0.04–0.13 mm; range SDC 0.11–0.36 mm) and sagDESS MRI (range SEM 0.05–0.10 mm; range SDC 0.13–0.29 mm; Table 6). SEM and SDC for cartilage volume and total area of subchondral bone are shown in Table 6.

**Table 6:** Standard error of measurement (SEM) and smallest detectable change (SDC) thresholds computed from year-1 and -2 follow-up MRIs of *n* = 21 knees from the test set

	corFLASH				sagDESS			
	Manual		U-Net		Manual		U-Net	
	SEM	SDC	SEM	SDC	SEM	SDC	SEM	SDC
*Cartilage thickness* (*mm*)								
MFTC	0.09	0.24	0.07	0.21	0.07	0.20	0.10	0.28
LFTC	0.10	0.27	0.13	0.36	0.10	0.29	0.10	0.28
MT	0.05	0.14	0.04	0.11	0.05	0.13	0.05	0.15
cMF	0.06	0.16	0.05	0.15	0.06	0.16	0.08	0.21
LT	0.06	0.15	0.09	0.26	0.08	0.23	0.06	0.18
cLF	0.06	0.17	0.08	0.21	0.06	0.16	0.08	0.22
*Cartilage volume* (*mm*^*3*^)								
MFTC	90	249	112	311	90	250	112	312
LFTC	98	273	153	424	118	326	175	484
MT	73	202	94	261	63	175	79	218
cMF	43	118	56	155	49	135	67	185
LT	83	231	113	314	100	277	120	332
cLF	43	119	79	219	41	113	89	247
*Total area of subchondral bone* (*cm*^*2*^)								
MFTC	0.17	0.46	0.44	1.22	0.34	0.93	0.59	1.63
LFTC	0.18	0.49	0.64	1.77	0.25	0.68	0.63	1.75
MT	0.13	0.37	0.32	0.89	0.25	0.68	0.42	1.17
cMF	0.12	0.32	0.25	0.68	0.14	0.38	0.38	1.06
LT	0.15	0.41	0.53	1.47	0.18	0.49	0.39	1.08
cLF	0.10	0.29	0.33	0.93	0.16	0.45	0.36	0.99

SEM and SDC were computed from coronal FLASH (corFLASH) and sagittal DESS (sagDESS) MRI acquired at the OAI year-1 and -2 follow-up visits

*MFTC/LFTC* medial/lateral femorotibial compartment, *MT/LT* medial/lateral tibia, *cMF/cLF* central medial/lateral femoral condyle

## Discussion

In this study, we have evaluated the segmentation agreement, accuracy, and the longitudinal test–retest reproducibility of an automated, 2D U-Net-based method for the segmentation and quantitative morphometric analysis of articular cartilage, using two MRI acquisition contrasts and orientations frequently used in clinical trials. The results demonstrate not only a high level of agreement of the segmentations, but also a high level of accuracy, and longitudinal test–retest reproducibility of morphometric analyses derived from the automated method, relative to those obtained from quality-controlled, manual segmentations as ground truth.

The U-Net architecture was chosen for automated segmentation, because it was designed to provide precise segmentations even when trained with relatively few examples [13]. The U-Net was originally intended for segmentation of neuronal structures in electron microscopic stacks, but previous studies have successfully applied it to segmentation of various musculoskeletal structures including cartilage [14–21, 23, 34]. The current study extends previous work on the relatively good agreement and accuracy demonstrated by U-Net-based cartilage segmentation methods [14–21, 23] by evaluating the accuracy, and particularly the longitudinal test–retest reproducibility of a U-Net-based segmentation pipeline for cartilage morphometry from two different MRI contrasts and orientations. This is a prerequisite before a segmentation technique can be applied to longitudinal MRI acquisitions from observational or clinical trials, with the main purpose of this technique being to detect small longitudinal changes in clinical trials and to measure the potential impact of disease-modifying treatment on these longitudinal changes. Recent studies also reported that the performance of the U-Net architecture for the segmentation of knee cartilages is on par with that observed for other current network architectures such as the V-Net, SegNet, and DeepLabV3+ [23, 37]. In contrast to the technique proposed by Ronneberger et al. [13], the current study did not employ data augmentation to artificially increase the number of examples for the training. This decision was based on the observation that data augmentation did not improve the agreement with manual segmentation results, when initially evaluating the impact of various parameters before the conduct of this study (data not shown). This observation was likely, because simple data augmentation techniques may not fully capture the heterogeneity of real-world data to improve the internal representations learned by the network. The same was observed when evaluating different loss functions (dice vs. weighted cross-entropy) or different weights for the weighted cross-entropy loss function, which were found to have a negligible impact (data not shown).

Similarly, we observed consistent results when repeating the training of the model using the same data, demonstrating the repeatability of the model training. Similar metrics were also observed when reverting the assignment of data to training, validation, and test set. The combination of features used for training the networks, in contrast, had an important impact on segmentation agreement: some combinations, such as including both the medial and the lateral femoral condyle in one model for sagDESS, did not lead to high segmentation agreement, most likely because of the similarity of the medial and lateral femoral cartilages. We, therefore, trained two separate networks for the segmentation of medial and lateral femorotibial compartment cartilages from sagDESS and this combination showed a similar performance as the one network trained for the cartilage segmentation from corFLASH MRI, despite the differences in orientation, resolution, and contrast. A combined network trained for the segmentation of both sagDESS and corFLASH MRI was also evaluated but showed a worse performance than the chosen combination of separate networks. It remains unknown, whether this is due to the different orientation or contrast (or a combination of both), but we conclude that sequence- and contrast-specific models may be superior to more general models that take greater variability of the features into account.

Most previous studies using CNNs for automated femorotibial cartilage segmentation reported DSCs between 0.78 and 0.92, and VOEs between 17 and 34% [14–16, 18–21, 23] and only one study using a combined bone and cartilage segmentation pipeline reported a higher DSC of 0.98 for femoral cartilage [17]. The agreement observed between automated and manual segmentations in the current study, therefore, compared favorably to that reported previously, both for corFLASH and sagDESS MRI. However, it should be noted that DSC comparisons across studies should be made with caution due to differences in which subjects the automated approaches are tested on.

The main purpose of the post-processing was not to improve agreement between both segmentation methods, but to correct implausible segmentations that precluded the computation of quantitative parameters of cartilage morphology. The post-processing step hence only had a small impact on overlap-based measures of agreement (DSC and VOE), whereas the distance-based measures (HD and ASSD) were improved substantially. This can be attributed to the higher sensitivity of distance-based metrics to implausible segmentations where the real boundaries of the cartilage are missed.

The automated segmentation produced consistently greater cartilage thickness of up to 5% than manual segmentation, with this systematic offset being more pronounced in corFLASH than sagDESS. Similar offsets were observed for cartilage volume, but not for the total area of subchondral bone, indicating that the overestimation is not caused at the edges of the cartilage plates but at the bone–cartilage interface or the articular cartilage surface. A similar overestimation of cartilage thickness and volume has also been observed previously for U-Net-based segmentations [14]. Yet, because these were consistent longitudinally, and because correlations with manual segmentations were high, this does not preclude that longitudinal changes in cartilage thickness (the main focus in clinical trials investigating the efficacy of therapeutic intervention) can be measured with the same sensitivity to change as by manual segmentation.

The current study relied on knees from the healthy reference cohort that were additionally confirmed to be free from radiographic OA. Some of these knees already had joint abnormalities visible on MRI [38] that did, however, not translate into a significant, disease-related change in medial or lateral femorotibial cartilage thickness over the first 2 years after enrollment, the period also included in this study [26, 39]. The statistically significant changes observed in the knees from the test set between year-1 and -2 follow-up for some of the measures can therefore most likely be attributed to statistical artifacts induced by measurement error. In addition, the changes were mostly comparable between cartilage measures computed from automated and manual segmentations, indicating a similar longitudinal reproducibility for both segmentation methods. This was also confirmed by the SEM, which was comparable for cartilage thickness computed from manual and automated segmentations. The precision errors observed in the current study were in the same range as those reported by Brem et al. and Tamez-Pena et al. for sagDESS [12, 40] and lower than the test–retest precision errors previously reported for corFLASH and sagDESS from unpaired, manual segmentations [24]. The low precision errors observed with the automated segmentation method is encouraging and advocates further application to longitudinal image acquisitions of osteoarthritic knees to evaluate its sensitivity to longitudinal change in cartilage thickness (cartilage loss). The test–retest precision errors in the test set were also not observed to be greater than those in the training or validation set. Rather, validation set test–retest errors of the automated segmentations were greater with corFLASH, because of implausible segmentations in a small number of knees. These findings suggest that the U-Net was not affected by overfitting to data used for the training process. At the same time, these findings highlight the importance of expert quality control, to ensure correct and accurate cartilage segmentations, and they highlight the challenge of applying fully automated segmentation blindly, without thoroughly checking segmentation results.

A limitation of the current study is that it only included radiographically normal knees from asymptomatic patients. However, approximately 50% of these knees demonstrated femorotibial cartilage lesions, along with other structural pathologies such as osteophytes, bone marrow lesions, meniscus damage and extrusion, effusion-synovitis and Hoffa-synovitis that affect either the cartilage appearance or that of surrounding tissues [38]. Still, these lesions did not translate into a detectable pathological change in medial or lateral femorotibial cartilage thickness over the first 2 years as previously reported [26, 39]. The OAI healthy reference cohort was, therefore, not only selected as a starting point in testing the U-Net-based segmentation approach, but also because the longitudinal reproducibility of the measurement can only be evaluated in the absence of pathological cartilage change. Hence, the OAI healthy reference cohort was ideally suited for that purpose, and has been previously used to establish progressor thresholds of cartilage loss [26]. Given that test–retest errors using the U-Net segmentation approach were similar to those from manual, quality-controlled segmentations, it can be assumed that the progressor thresholds for cartilage thickness change also apply for automated segmentations. Another limitation of the method is that, although the U-Net provided accurate segmentation for many of the knees, it failed to provide complete cartilage segmentations in some of the slices, and produced implausible segmentations in others. We were able to overcome some of these errors using the post-processing steps, but a simple, rule-based approach cannot compensate for incomplete segmentations. Such incomplete segmentations are most likely explained by the fact that the U-Net has no “real” knowledge about the context of the cartilages, and none about valid shapes. We, therefore, strongly recommend thorough quality control of all segmentations by an expert reader, and to perform manual corrections of automated segmentations where needed. Another limitation of the current study is that the femoral ROI marked by the readers in the manually segmented data sets was applied to both the manual and the automated segmentations to ensure comparability between manual and automated measures. This femoral ROI was, however, necessary to exclude posterior parts of the femoral cartilages from the segmentation, which are affected from partial volume effects in coronal MRIs and display a lesser amount of longitudinal change than the weight-bearing part in knee OA [30]. A strength of the current study is that it did not only confine itself to the analysis of DSCs and other measures of segmentation similarity, but also directly evaluated the accuracy and longitudinal test–retest reproducibility of morphometric cartilage measures, such as thickness, volume, and surface area derived from the automated segmentations. Another strength is that the approach was tested in the same knees for two different MRI contrasts and orientations, which are both frequently applied in clinical trials. Finally, the current study provided progression thresholds based on the SDC methodology [36], which can be used for classifying knees into those showing progression vs those who do not show progression.

In conclusion, this is the first study to test the accuracy and longitudinal test–retest reproducibility of quantitative cartilage morphometry using an automated, U-Net-based segmentation approach, using the two image contrasts and orientations that are most frequently used in clinical trials. We not only demonstrate a high level of agreement between automated vs. manual “ground truth” segmentation, but also a high level of accuracy, and longitudinal test–retest reproducibility for morphometric analysis of articular cartilage derived from the automated method. Yet, post-processing steps and expert quality control are highly recommended. Future research will establish with which level of sensitivity the method is able to detect longitudinal change over time in diseased knees, and the efficacy of therapeutic intervention on stopping or reverting articular cartilage loss in osteoarthritis.
